# A place to grow: Well-being and activism on Edinburgh’s post-war allotments and how this can inform urban gardening in Scotland today

**DOI:** 10.12688/wellcomeopenres.15216.1

**Published:** 2019-04-17

**Authors:** Hannah Connelly

**Affiliations:** 1 Department of History, University of Glasgow, Glasgow, UK

**Keywords:** Community, Allotments, Community Gardens, Well-Being, Health, Post-War, City-Planning, Scotland

## Abstract

Between 1945 and 1970, allotments which had been established in Scottish cities during the Second World War were removed by local authorities to build new housing, schools and playing fields. This was a crisis for the plotholders, who had formed communities on the allotments and found gardening to be highly beneficial to their physical and mental health. This article takes an archive-driven approach using the history of post-war allotments as a direct parallel to community gardens in Scottish cities today, which are often praised for their positive outcomes in local communities for health and well-being but are frequently only temporary, established precariously on land earmarked for development. The article argues that for urban gardening to be truly beneficial for health and well-being, permanence is needed to remove the negative stresses of possible closures.

## Methodology

This archive-driven research article has been written using the collection *The Papers of Victor Webb* (GB248 UGC 222) held by University of Glasgow Archives and Special Collections, as well as primary sources from Edinburgh City Archives and Glasgow City Archives ^1^. The collection, deposited by the Scottish Allotments and Gardens Society (SAGS), incorporates the records of SAGS and the Scottish Allotments Scheme for the Unemployed, which were collected by Victor Webb, an Edinburgh plotholder greatly involved with both organisations. In 2017 the collection was made publicly accessible after cataloguing funded by the Wellcome Trust Research Resources scheme; the catalogue is available from the Jisc Archives Hub. This article puts forward historical case-studies as an opportunity for discussion in regards to present forms of urban gardening and its associated benefits for health and wellbeing.

## Introduction

During the Second World War the Scottish allotments movement gained momentum as new plotholders took on the challenge to *Dig for Victory*. The government’s promotion of allotments became a key part of the strategy for the home front to manage extreme food shortages, cementing a link between allotments and healthy eating ^2^. However, it is the history of the allotment movement in the post-war period that closely parallels issues facing urban gardeners today. Wartime allotments were often established on land already earmarked for development, which had been delayed by labour and supply shortages during the war; when the war ended this land was reclaimed. For plotholders this was a crisis; their allotments had become their communities. This offers a direct analogy to Scottish cities today, particularly Edinburgh and Glasgow, where residents are encouraged by both local authorities and charities concerned with housing, health and wellbeing, to create community gardens for outdoor exercise, fresh food and social cohesion on land designated for developments that have been stalled by economic recession ^3^. Although the health benefits of community gardens have been well researched, there has been little comment on how closures—when the developers are ready to work on the land—affect the gardeners involved. This article will use the similarities of allotment closures in the post-war period to consider this.

Today, plotholders and community gardeners claim ‘growing your own’ makes fresh fruit and vegetables more accessible, yet community gardens are not provided with the longevity vital for planning crops. Scotland has one of the worst health profiles in Western Europe, due in part to poor diet and lack of exercise, yet the potential of urban gardening as a solution is not being fully investigated ^4^. This article will not provide that investigation—that issue is beyond its scope—but it will examine an historical case study to gauge whether allotments and community gardens planted in temporary spaces can help improve the health and well-being of Scotland’s urban population.

This article will investigate the closure of allotments between 1945 and 1970, a period which saw SAGS members becoming activists fighting to save their sites, in order to answer three questions: How did SAGS campaign for the survival of allotments in the post-war period and how did this shape the development of the society?; what was the impact of allotment closures on plotholders in the post-war period and how can this impact inform the use of community gardens today?; and, is there a need for permanence on allotments and community gardens to truly benefit health and well-being? The article will move from a history of the Scottish allotment movement in Edinburgh from 1945 to 1970 to a case-study of a site closure and conclude with a discussion of what this could mean for community gardens today. Through answering these questions, this article will argue that the insecurity of tenure, the dark shadow of closure, of allotments in the post-war period caused great stress to plotholders, therefore negating the positive experiences they could have gained from urban gardening—the prevention of loneliness, improvement of both physical and mental health and access to healthy food. The article will argue that we must learn from the past and provide better protection for present allotments and community gardens in Scottish cities so that urban gardening can truly benefit both individuals and local communities as a whole.

## The Scottish Allotment Movement, 1945–1970

The Scottish Allotments and Gardens Society was constituted in 1946, merging the Scottish National Union of Allotment Holders (founded in 1917) with the Department of Agriculture for Scotland’s wartime allotments committee. SAGS provided various services—insurance, supplies of fertiliser and typing—but its major yearly event was an annual conference which enabled the voluntary committee to disseminate information and other members to join their discussions. The Society had government funding for a full-time secretary ^5^.

The first secretary, William McWilliam, wrote to members that the main aim of SAGS was ‘to fight for the establishment of PERMANENT ALLOTMENTS in all large cities and to help and assist all amateur gardeners in their work of home food production of vegetables and horticulture’ ^6^. The closure of allotments for housing developments had begun even before the Second World War was over, despite continuing food shortages, so the new society began its campaigns to protect allotments from the beginning. Although Scotland had suffered little air-raid damage, with the terrible exception of Clydebank, the country’s housing stock had still been affected by labour and supply shortages, delaying repairs and suspending new developments. At the end of the war there were 120,000 houses to be replaced immediately in Scotland. The government had classified 200,000 as overcrowded; 405,000 had no sanitary conveniences or internal water supplies and 64,000 had been damaged during the war. In addition, 134,000 new households had formed and needed homes ^7^. In 1947 SAGS invited the Under-Secretary of State for Scotland, Robert Fraser, to give an address at their annual conference on ‘the vexed question of security of tenure’. Fraser said that allotments were indeed being taken for new housing, schools and industrial development but the closures were also due to a lack of interest as Scottish people were not ‘sufficiently vegetable conscious’—there was no interest in growing vegetables now that the war was over ^8^. Having heard such an opinion from a government representative, the society realised allotments would need further support and protection. Following the conference they sent a letter to all Scottish MPs asking for new legislation to give plotholders better security of tenure ^9^.

In January 1948 SAGS organised a meeting on food production with two hundred representatives from local authorities and allotment associations ^10^. Arthur Woodburn, the Secretary of State for Scotland, was invited to give a speech but it must have severely disappointed the plotholding delegates when he stated that when it came to a choice between housing or allotments, ‘the allotment holder would have a hard time of it’ ^11^. Following this meeting SAGS circulated a memorandum to Scottish MPs, again asking for a revision of existing allotment legislation ^12^.

SAGS could not argue that allotments were more important than housing; it was clear that Scotland needed new homes. However, plotholders did believe, from their own experience, that allotments were needed alongside housing, especially flats that had no personal outdoor space. Wartime allotments were not failing because Scottish people were not interested in gardening but because sites were unplanned and placed on any available land. This meant that plots were too far away from people’s homes, the soil was often too poor for cultivation and there was a lack of fencing, allowing vandals to easily enter sites. McWilliam wanted to see allotments properly integrated into town planning to correct these issues. He also believed the plotholders themselves needed to take responsibility for protecting allotments. In the 1948 annual report, he requested that SAGS members wrote to their candidates in the local election asking for their views on allotments. He also asked them to keep their plots ‘clean, tidy and properly cultivated’ as a tidy plotholder could not be accused of being uninterested ^13^. This request for tidiness was later to become key to the allotment movement’s campaigns to protect sites.

The SAGS campaigns for new legislation appeared to work out well and the Allotments (Scotland) Act was passed on 26 ^th^ October 1950. However, the society’s celebrations were short-lived. What McWilliam and the committee had not anticipated was that on the same day the Housing (Scotland) Act was also passed with a clause that took legal precedence over a key clause in the allotment legislation, in effect cancelling it out. The 1950 Allotments (Scotland) Act stated that if allotment ground was to be reclaimed then one year’s notice had to be given to allow crops to be harvested; if shorter notice was given then compensation had to be paid ^14^. This was not full security of tenure as SAGS had campaigned for but it did improve conditions and at least gave some protection against the financial worries of being dispossessed from a plot. However, Section 63 of the 1950 Housing (Scotland) Act stated that a local authority that had purchased land for providing houses could enter and take possession within 14 days ^15^. This did not just apply to newly purchased land but also land that had been originally purchased for housing and then used temporarily as allotments during the Second World War; so, if an allotment site was required specifically for new housing a local authority need only give two weeks’ notice to plotholders, not a full year.

It did not take long before such an incident occurred. In March 1951 the West Mains allotment association in Edinburgh received a letter from the Depute Town Clerk informing them that their site was to be used for a housing development, schools and playing fields. In total, 55 of the 214 plots on the site would be affected and the plotholders had only been given 14 days’ notice, in accordance with the provisions of the 1950 Housing (Scotland) Act ^16^. The plotholders were shocked. Two years previously they had been assured by the Edinburgh Garden Allotments Committee that their tenure was permanent and again in November 1950, when they had sought confirmation after viewing the City Development Plan, they had been told that their site was permanent ^17^. McWilliam wrote to *The Scotsman* newspaper on 2 ^nd^ April 1951 stating that West Mains should be ‘a warning’ to all plotholders in Scotland ^18^.

A few days later, a member of the SAGS Committee, W.W. Simpson, also wrote to *The Scotsman* stating that the dispossession of the plotholders at West Mains showed ‘a deplorable lack of sympathy by the Local Authorities for the needs and importance of the allotment movement’. Simpson wrote that not only were allotments being closed for housing, such as at West Mains, but also playing fields. He criticised local authorities for not realising that they had a duty to provide permanent allotments and to put sites ‘into a neat and tasteful condition in the same way as they set aside and lay out a piece of ground for recreations such as football, cricket, bowling or tennis’ ^19^. Simpson’s letter marks an important change of direction in the allotment movement. Allotments were no longer regarded by local or national government as a necessity so SAGS’ arguments based on food production were simply not working. A new argument was needed and Simpson was putting forward that allotments were not just for food but for recreation, with the physical and mental health benefits recreation provides. On 9 ^th^ April 1951 McWilliam and the secretary of the Edinburgh Allotment Holders’ Association (EAHA), Andrew Judge, wrote to the Secretary of State for Scotland—Hector McNeil—also stressing the benefits of allotment gardening as a recreation for ‘promoting the health and resources of an urban community in peace-time’ ^20^. The MP for Edinburgh South, Sir William Darling, showed his support by writing to the Edinburgh Town Clerk and McNeil, particularly commenting on the benefits of allotments for people who live in flats ^21^. Unfortunately the campaigning efforts of SAGS, the EAHA and the plotholders themselves could not save the affected plots at West Mains. Judge received a letter from the Depute Town Clerk stressing the need for new houses and stating that ‘the interests of the allotment holders must be subjugated to the interests of the community as a whole’ ^22^. For the plotholders, the closure of their allotments would have been devastating. They had been aware that closure was a possibility, that is why they sought assurance upon seeing the City Development Plan, and the high level of their campaign—even attempting to involve the Secretary of State—demonstrates the energy they put into saving their site. Apathetic plotholders simply would not have tried; campaigns need time, effort and emotional input.

At the 1952 SAGS conference one of the dispossessed West Mains plotholders, Victor Webb, gave a talk emphasising how vital allotments were for recreation for people of all ages. He said allotments could fill the elderly’s time and keep them ‘free from minor ailments’; they were a place for young people to get ‘rid of their high spirits and energy’ and for everyone allotments meant ‘fewer troubles, breakdowns, fewer cases for the mental homes, fewer matrimonial disputes’. Allotments, according to Webb, also allowed for mixed income groups and ‘people from all walks of life to come together’. He asked plotholders to write to their local councillors and MPs to persuade them to include allotments in city development plans ^23^. This request was repeated by McWilliam in the 1952 annual report when he requested plotholders to study their city’s development plans and lodge any objections with the Secretary of State for Scotland. Again he stressed the importance of allotments in tenement areas: ^24^

“While we admit gardens are being supplied in housing schemes, these do not meet the necessary requirements of tenement dwellers who desire allotments in or within easy reach of their homes where they can follow up a healthy and recreational hobby while assisting in the vital necessity of home food production.” ^25^

The SAGS message was increasingly that allotments were for recreation, particularly for people living in inner-city flats and tenements. Although rationing was still in force in 1952, government priorities had changed and SAGS needed a new argument to protect allotments. Both Webb and McWilliam continued to mention food production, even stating that it was ‘vital’. The allotment movement recognised a new argument was needed to win over the government but amongst plotholders growing fruit and vegetables remained an essential part of having an allotment. The Society was becoming increasingly concerned by the loss of allotments in Scotland’s larger cities. In 1954 they invited the Joint Under-Secretary of Scotland, W. McNair Snadden, to give a talk at their annual conference. Snadden stated that there had been 20,000 allotments in 1939, 83,000 in 1943 and the number had reduced again by 1954 to 22,000. However, he thought that the new housing estates made up for any loss of food production and said it could be expected that fewer people wanted allotments now that food shortages were over ^26^. So there was little support from national government for the allotment movement and local authorities continued to close sites for housing developments.

In Edinburgh there was a shortage of 10,000 houses in 1945, which led to a rapid development of public housing throughout the 1950s and 1960s ^27^. This affected both permanent allotment sites, such as West Mains, and wartime allotment sites, such as Ladywell Road in Corstorphine, which was removed by the Housing Committee in March 1955 ^28^. Although gardens might have been provided in the new housing development, plotholders would not necessarily find new homes there themselves. Landowners whose ground had been requisitioned during the war were also asking for their land to be returned to them; for example, a lease for emergency allotments at Robb’s Loan ended in December 1955 and 33 people lost their plots with only room for 17 of them to move to nearby sites ^29^. Where they could, Edinburgh Corporation (the local authority before the City of Edinburgh Council was created under the 1973 Local Government (Scotland) Act) did try to keep allotments open. The wartime allotments on the Meadows were to be closed in November 1952, but the Corporation saved a site on an area known as the Archers’ ground and in a ‘triangular area’ for a further five years. Of the plotholders there, 209 applied to keep their plots but only 143 were available. Preference was first given to plotholders whose own plots were being retained, then to old-age pensioners and then to plotholders ‘who had kept their plots in good condition’, showing that SAGS had been right to stress the importance of keeping plots tidy ^30^. Andrew Judge had also picked up on this and wrote to the members of the EAHA in August 1955:

“Plots should be properly worked and adjoining paths kept clean. This will no doubt affect our security of tenure and, in the interests of us all, the Committee insists that plots be properly worked, paths kept clean and huts, toolsheds etc. made respectable.” ^31^

The Edinburgh Corporation does seem to have based many of its decisions on whether or not allotments were ‘tidy’. In November 1955, Liberton and Craigmillar Estates wrote to the Corporation asking for their requisitioned land on Liberton Brae to be released back to them, but as the plots were all occupied and ‘in very good condition’ the Corporation reached an agreement with the landowners to keep the site ^32^. In 1957 the Corporation bought a site at Saughton Mains from its landowners as they considered the 184 plots there to form ‘one of the best allotments in the city’ ^33^. Conversely, in January 1956 the Corporation decided to clear a site at Niddrie Mains as they considered the plots to be in ‘a completely unsatisfactory condition’ ^34^. The problem was that the reasons plots were untidy were not always taken into account; it was not always because of a lack of interest. In September 1955 the Corporation decided to serve notice on plotholders on a site at Bruntsfield Links after receiving complaints from local residents about their condition. However, these plotholders had been warned that their site was possibly going to be used for a new building development and they had therefore not put the effort into their plots that they might usually have done and the site had deteriorated ^35^.

By the mid-1950s allotments lost to housing developments had become a constant theme of SAGS’ annual reports. Reginald Ashley, who became the secretary after McWilliam’s death in 1953, argued that it did not matter that new houses had gardens as those who lived in tenements were left without allotments ‘depriving our members of a healthy and interesting recreation’ ^36^. Following the 1956 conference, Ashley sent a resolution to the Department of Agriculture for Scotland asking for further protection for allotments and a change in legislation. The Department replied stating that they felt a general directive to local authorities would be more effective but as they had already sent a directive in April 1955 stressing the importance of allotments, they were unwilling to do so again ^37^. By 1959 Ashley was angered by the lack of government support. In his opinion, allotments were disproportionately targeted by planners and developers to other recreations and he felt let down when he considered how supportive plotholders had been to the government during both World Wars:

“When convenient, memories can be very short, for within living memory there has been two world wars, and during both periods the allotment holder was considered a very important person by the National Government and the Local Authorities and as he did not fail the country in the time of emergency, surely he is justly entitled to expect a square deal in times of peace.” ^38^

However, throughout the 1960s sites continued to close. In 1960 Ashley used the annual report to comment on the loss of sites to both private builders and local authorities. He attributed the loss of many to rumours that the sites were going to be closed, which led to so much uncertainty that plotholders either did not work their plots fully or gave them up and associations disintegrated ^39^. This then made the sites appear neglected and local authorities could justify their actual closure. Despite their site being zoned as allotments in the Edinburgh City Development Plan, plotholders at a site on Hamilton Drive were under the impression that their site was to be developed and so there had ‘been a deterioration in the conditions’ there. This led to the Depute Town Clerk writing to the Secretary of the Scottish Development Department in April 1964 to say that the site should be developed for residential purposes, even though he wrote that he understood that the lack of cultivation on the plots was ‘due to a feeling of insecurity of tenure’ ^40^.

Both tidiness and deterioration on plots can be regarded as a sign that plotholders were extremely worried by the uncertainty of whether or not they could remain on their allotments. SAGS and the EAHA called for tidiness on plots to demonstrate that they were fully cultivated and actively in use, but this would have restricted differences in gardening methods and did not allow for temporary difficulties for plotholders such as an illness or a new baby. Conversely, messiness can be the result of a plotholder concerned about security of tenure and unwilling or unable to take the financial or time-consuming risk of cultivating a plot even if it is wanted. Tidy sites were more likely to be saved by the Edinburgh Corporation, which meant sites in wealthier areas might have been more likely to be saved where this risk could be taken without fear of financial loss.

In December 1964 the Superintendent of Parks and the Chairman of the Garden Allotments Committee wrote a review for the City Development Plan, based on visits to 10 sites. Their main concern during this review also seems to have been tidiness; for example, they commented that the temporary site in Edinburgh Valley Park ‘always looks untidy and naturally gives this otherwise neat and tidy park a bad name’ ^41^. This followed a review of allotments in February 1963 by the Civic Amenities Committee, for the quinquennial review of the 1957 City Development Plan. The plan had suggested 15 acres of new allotment sites, to be included in new housing areas and to replace existing allotments that were to be closed for new developments; however, the 1963 review stated that 15 acres had ‘not proved to be practicable’. They had found that there were 110.30 acres of allotments in Edinburgh, including 1508 Corporation plots, and unsurprisingly the greatest demand for these was in densely populated areas and the lowest where gardens were provided with new houses. However, the Committee expected that overall demand would continue to fall as people were attracted to ‘new leisure activities’ ^42^. After completing their review, the Superintendent and the Chairman suggested there should be at least 10 acres of permanent allotments in Edinburgh, provided with communal huts, toilets, standardised greenhouses and sheds, paths, fencing and water supplies. Such facilities match with the allotment movement’s own calls for allotments to be treated as other recreational sites, but whereas SAGS called for permanent sites to be planned and created within cities, for people living in tenements and flats, the Superintendent and the Chairman wanted to create permanent sites in the green belt surrounding the city, to keep the ‘unkempt appearance of allotment areas’ away from the parks ^43^.

SAGS were also carrying out their own review of allotment provision. In 1962 they wrote to local authorities in England and Wales and found that amenities were generally better but charges were higher. In a separate survey of Scotland they found that ‘on the whole’ local authorities provided better facilities than private sites and, as the Edinburgh Corporation had also found, that the greatest demand for plots was ‘in crowded tenement areas of our cities’ ^44^. SAGS believed the future of the allotment movement to be reliant on local authorities as they had control over land:

“With the value of land soaring to such astronomical heights it is very obvious that the future of the Allotment Movement in Scotland, especially in our cities and large towns, lies with the local authorities in the land they control on which has been provided statutory allotments, for such land if properly cared for and cultivated will remain the heritage of the tenement dweller for many years to come, for most local authorities realise that they have a duty to provide, where possible, the town dweller with the necessary facilities for the pursuit of the recreation that the cultivation of an allotment affords.” ^45^

Ashley’s phrase ‘the heritage of the tenement dweller’ is key; he was intrinsically linking allotments with the idiosyncratic architecture of Scottish cities, cementing SAGS’ argument that allotments needed to be integrated into city planning. Unfortunately not many Scottish local authorities agreed with this view. Before their 1964 annual conference SAGS held a separate meeting with 16 local authority representatives. They had little sympathy for SAGS as they believed that allotments were just not wanted; allotments provided around new multi-storey flats in Dunfermline had not been taken up; the Superintendent of Parks from Paisley said many of their plots were vacant; and the representative from Rutherglen said that people in new houses did not want allotments in addition to their gardens ^46^. In 1965 they held another pre-conference meeting but again the local authority representatives said they were closing allotments because of a lack of interest. Ashley argued that for allotments to be successful they needed to be put on the same level as other recreations: ‘Our aim must be to get the allotment movement recognised at all levels as a means of providing a healthy recreation and one that can be enjoyed by people of all ages’ ^47^.

To help with their campaigning SAGS began to consider more closely what the purpose of an allotment was and who was using them. They sent out a survey to plotholders in 1965 and discovered that there were a ‘hard core of enthusiasts’ around Edinburgh, Glasgow, Dundee and Aberdeen. The average age of a plotholder was between 40 and 60 and there had been ‘an increase in the numbers of professional men and white-collar workers cultivating the soil’. Many sites were full with waiting lists but others had vacant plots. This was mainly because of lack of security of tenure but there was also competition from motorcars, bingo and television ^48^. The results of the survey made it even clearer to SAGS that allotments were no longer considered a necessity and that the future of the movement needed to be in recreation. Ashley thought a name change might be useful, for example, to ‘Leisure Garden’ ^49^. SAGS also felt that the definition of an allotment needed to be updated; allotments were no longer vital for food production and people were easily buying vegetables, either fresh or tinned, from supermarkets ^50^.

Like SAGS, the Federation of Edinburgh and District Allotments and Garden Associations (FEDAGA) had become convinced that the future of allotments lay in recreation as ‘leisure gardens’. FEDAGA wrote to the Civic Amenities Committee in May 1968 suggesting that allotments could be based on the European model: ‘many devoted to ornamental plants, some with pleasant chalets or week-end huts attached so that whole families can enjoy the delights of gardening’ ^51^. They argued that it was a good time to consider modernising allotments by providing proper roads, water points, fencing and huts, and if the Corporation provided these things, then rent could be increased in keeping with the new facilities. FEDAGA felt that insecurity of tenure remained the ‘primary factor’ for neglected allotments as plotholders would not put enough time and money into their plots without knowing they would be there for more than a year at a time ^52^. In parts of the Corporation, FEDAGA found support. The City Architect agreed with their suggestions and added that a central grass reservation could be added to sites for children to play on. The Director of Parks and Recreation saw that FEDAGA’s ideas fitted with the Civic Amenities Committee own for sites within the green belt with modernised facilities. He also wanted to have better security of tenure for these sites, perhaps securing an agreement with the Town Planning Department that it should not be used for any other purpose for 15 to 20 years. Dundee’s local authority had already created a model site, providing plots with greenhouses and electrical points and the Director thought Edinburgh could take inspiration from this ^53^. However, both the Estates Surveyor and the Depute City Chamberlain were unsure whether plotholders would really be willing to pay an increased rent ^54^. Without full support within the Corporation, the plans for permanent sites in the green belt did not materialise.

Such plans for permanent, recreational allotments were also suggested in the *Thorpe Report*. In 1965 Harry Thorpe, the Head of Geography at the University of Birmingham, was appointed to lead a government committee reviewing allotment policy in England and Wales. His report, published in 1969, showed that the relationship between the allotment movement and local authorities was ‘at a lower ebb than any time in the movement’s history’; Thorpe believed there was a strong possibility that allotments would completely disappear ^55^. This was not something Thorpe wanted to see happen. Like SAGS, he argued that allotments were good for both physical and mental health; they enabled creativity, provided fresh food free from pesticides and fertilisers and that they promoted ‘a strong community feeling’ ^56^. He advocated the creation of chalet gardens in city peripheries, similarly to the Edinburgh Director of Parks, but he also argued for gardens within high density housing for those who could not travel easily or had little spare time. Also like SAGS, Thorpe wanted to see the gardens receive equal consideration in development plans to other recreational facilities and given full security of tenure ^57^. Although the report was only for England and Wales, it gave SAGS hope that if Thorpe’s recommendations were implemented across the Border, they would soon follow in Scotland. As it happened, none of his suggestions were acted on by the government ^58^.

This was despite others beyond the allotment movement recognising their usefulness. Thorpe put a strong emphasis on the communal nature of allotments arguing that the gardens formed ‘bonds of companionship and co-operation’:

“…the benefit which allotment gardens provide to those who are lonely or live alone and to retired people who may have no other incentive to induce them to mingle with their fellows, may be so great as to create an argument even for providing allotments *instead of* home gardens in certain cases.” ^59^

This appealed to social researcher Pearl Jephcott in her 1971 study of multi-storey life in Glasgow, *Homes in High Flats.* Jephcott was highly critical of multi-storey flats and wanted to see them discontinued as a form of social housing; she argued they were costly, negatively affected social life, exposed children to risks and did not save space unless open-space provision was ignored. Unlike life in tenements, multi-storey life had led to many families becoming isolated and was particularly difficult for young children and their mothers ^60^. Jephcott put forward Thorpe’s idea as an antidote to this loneliness, that gardens could be included around the bases of tower blocks to allow freedom of expression, provide ‘solace for the lonely’ and give interest to the retired ^61^.

Providing space, not only for private gardens with individual homes but also for community gardens or allotments for people in multi-storeys or high density housing areas, could have relieved some of the social problems later recognised in post-war developments. Pat Rogan, who was the Chairman of the Edinburgh Corporation Housing Committee in 1962, commented in 1997: ‘In retrospect, many improvements could have been made on the housing crusade of thirty years ago…many new housing estates were deprived, at the beginning, of amenities that would have made life more comfortable.’ ^62^ Gardens could have been such an amenity.

After the Second World War, SAGS adjusted their approach to campaigning for the protection of allotments in Scottish cities, arguing that the gardens were for recreation rather than the necessity of growing food. Government, both local and national, no longer recognised the need for allotments; wartime food shortages were over and recreation on allotments was not regarded as important as the construction of new housing stock within city boundaries. Researchers Thorpe and Jephcott recognised that recreation—including in the form of gardens—should have been a vital part of new housing developments for the prevention of isolation and loneliness that occurred without social facilities for residents. Instead the opportunity was missed. SAGS also wanted to see allotments included as part of city planning but the Edinburgh Corporation wanted to move permanent sites out to the green belt. This would have made allotments a privileged hobby; plotholders would need to drive or be able to afford regular bus fare. The formation of community gardens in Glasgow and Edinburgh today shows that there is still a demand for recreational gardening within cities, just as SAGS’ campaigns had advocated. If existing allotments had been protected or new allotments created in the post-war period, residents in areas of high density housing could have benefitted from the positive impacts of gardening on their physical and mental health, as described in present literature on community gardens. Instead, we are only just beginning to recognise the benefits of urban gardening and community gardens still remain in a precarious position.

## A case-study: St. Leonard’s allotment association

One of the difficulties in allotment history is that it is not always possible to see what plotholders themselves thought or felt about site closures. Most information comes from SAGS, local authority records and statistics. The following case-study of St. Leonard’s allotment association’s campaign to save their site aims to include the voices of plotholders and especially their fear of losing their site. St. Leonard’s was a permanent allotment site established in 1926, also with an area of wartime plots, situated on Salisbury Green in Edinburgh just off Dalkeith Road. The site is now occupied by University of Edinburgh student accommodation. The case study shows the attachment plotholders form for their allotments and their gardening community and the stress caused by lengthy periods of uncertainty over site closures. A place that had become vital to the plotholders’ health and well-being was under threat and they were desperate to be able to keep it.

In early August 1949, the Secretary of St. Leonard’s Allotment Association, William Smith, on Salisbury Green, wrote to Edinburgh’s Depute Town Clerk, Mr A. Walker, after his plotholders had been met with a worrying surprise: ‘…little metal posts with a piece of red tape fixed on them sticking on their plots. These posts are the kind used by surveyors’. One of the members had then met a surveyor on the site and had been told ‘it was being measured for playing fields’ ^63^. The Association managed to find out what was actually happening through a plotholder’s son who was studying architecture with a surveyor working on the site; a hostel was going to be built with playing fields and the plotholders would probably get a year’s notice to quit in about three years’ time. Smith wanted to know if the Corporation were aware of this ^64^. The Superintendent of Parks had not heard anything about the planned development either and wrote to the Depute Town Clerk that the Salisbury Green allotments ‘are probably the best in the city and an alternative site for the plotholders would be very difficult to find’ ^65^. The Corporation received notice in October 1949 to remove the allotments from the site by 31 ^st^ December 1949 from the solicitors of the University of Edinburgh, who owned the land ^66^. Because the development was not for housing the Town Clerk was able to have the notices withdrawn and extended, giving the Corporation and the plotholders until 30 ^th^ April 1950 ^67^. After meeting with representatives of the university, the Corporation’s Garden Allotments Committee managed to secure the site until November 1950 and Walker wrote back to Smith asking him to delay talking to the plotholders until the Garden Allotments Committee had had chance to consider their position ^68^.

By the end of July 1950, Smith had still not heard back from the Corporation so he wrote to Walker for an update; he wanted to know if he should be placing his usual orders for lime, manure and fertiliser. Smith’s letter demonstrates how difficult it is to manage an allotment site if you do you not know how long you will be there:

“If it were possible at all to get some sort of guarantee on the lines say of year to year we would be much happier and better able to make our arrangements for supplies, whereas, at the moment, we are a little scared to sign contracts for supplies which, for the good cultivation of the ground we must get each year.” ^69^

The uncertainty was beginning to distress the plotholders and they were leaving: ‘This groping in the dark is upsetting our members and in some cases, interest is failing, and it is very hard work on the part of my Committee to keep the interest going’ ^70^. Smith’s letter prompted Walker to write again to the University solicitors who agreed to extend the notices by one year but this did not satisfy Smith who asked Walker not to send the notices until he had found out from the University whether this would be the final warning:

“It is getting rather awkward to our members to be put into these quandaries, first, we were to get out last April, then it was extended to November, and in August last, the notice was delayed for a year - to November 1951.” ^71^

However, the university continued to extend notices by a year or two at a time, and when Smith sent a letter in April 1953 asking whether the plotholders should plant out their winter crops, the notice was extended again until January 1955 ^72^. Although this was good news, it still left the plotholders with worries that their site would eventually close, so in July 1953 the plotholders stepped up their efforts to keep their site open. They wrote to the Secretary of State for Scotland, James Stuart, protesting against the closure of the Salisbury Green site, which had been allotments for 37 years and had 127 members. They stated that the site was in ‘the most densely populated area in the city’ and that apart from the crops grown the plots contributed ‘in no small measure to the health and recreational and social welfare’ of not only the plotholders but also their dependents and children: ‘We are fully aware of the need of houses for the people, but the small area gained for this purpose does not justify the loss of an open lung in such an overcrowded district.’ ^73^

The Association also lodged an objection to the City Development Plan, in which Salisbury Green was zoned as residential ^74^. The objections to the plan in this area were fairly complicated. Although the University planned to build halls of residence on the land, which would count as residential, they had a disagreement over population density; the Corporation proposed a density of 140 people per acre but the University wanted it reduced to only 40 to 50 ^75^. The Town Planning Officer was against both the allotment association and the University’s plans for development. Although he agreed that the 127 plotholders and their dependents did benefit from the use of the land, he argued that ‘the development of the area as envisaged in the Development Plan’ would provide accommodation for 770 people who would ‘enjoy the exceptional amenities of the site’. Keeping the allotments would require a housing development elsewhere which would mean a loss in agricultural land and a ‘spread of the city’ ^76^. The Garden Allotments Committee agreed not to support the Association’s objection, however, the Superintendent of Parks remained supportive of ‘the best permanent allotment area in the city’ and the Committee searched for an alternative site ^77^. Meanwhile the University’s solicitors extended the notice period to 28 ^th^ January 1956, whilst they waited for the final decision from the Secretary of State on the population density for the area ^78^.

Smith wrote to his plotholders in October 1954 asking for applications for a move to a new site at Cameron Toll, on the Inch Estate on Old Dalkeith Road. The Garden Allotments Committee was in the process of creating new plots there which would be ploughed and ready for them and their security of tenure ‘would be made absolute’ ^79^. Smith received 71 applications but the Garden Allotments Committee had only planned for 40 plots ^80^. By October 1955 nothing had happened and Smith became worried about timing; the plotholders needed to leave their existing site by the end of January 1956. Smith asked Borland, the Depute Town Clerk, whether the plotholders could stay on Salisbury Green until the Secretary of State had made a decision or whether the Corporation could find a site that would accommodate everyone who wanted to move ^81^. The plotholders really wanted to stay on Salisbury Green. The new site was not big enough for all of them and they also considered it to be too far away for the elderly members to get to; moving to a new site would have fractured their close community. ^82^. The plotholders had a strong tie to their existing site and were unwilling to leave whilst there was still any chance the site could be saved:

“The Association were very reluctant to sever their connections with the area at this stage having in mind the possibility that the Secretary of State’s decision might preclude its use for any other purpose than allotments.” ^83^

The Town Planning Officer thought that by delaying their move the plotholders would prejudice their position for a new site as the Superintendent of Parks was going ahead with plans for 67 plots at Cameron Toll and these would go to new plotholders if the Salisbury Green plotholders resisted moving ^84^.

Victor Webb, who had become involved with helping other sites after his experience at West Mains, organised a meeting between the Administrative Assistant, R.M. Young, at the University and the President and Vice-President of the allotment association on 30 ^th^ November 1955 ^85^. Young offered that the plotholders could stay until the University had a decision from the Secretary of State. Once that was done, if it was in the University’s favour, there would be at least three months before the University could make a start on the development. The tenancy of the Corporation would still cease on 28 ^th^ January 1956, as agreed, but the Association would then be tenants of the University with no rent charged and no legal obligation on either side. The Association were happy with this arrangement ^86^.

The Secretary of State’s decision was in favour of the University. The first stage of building would take three years and affect four plots. After that the part of the site designated as a wartime ‘emergency area’ would be developed and it would then be 15 to 20 years before development in the permanent area would begin. William Smith wrote to Victor Webb that the plotholders were very happy with this arrangement:

“This information has given our members quite a new spirit, they have an idea where they are now and I can see Salisbury Green getting a right good clean up and repainted which it badly needs. We carry on as we arranged as the end of last year, no rents, no legal requirements, but quite obvious, on the most friendliest of terms on both sides.” ^87^

However, by February 1964 (less than eight years later) the St. Leonard’s plotholders had received notice from the University that the land would be required by the early autumn; they were left hoping that the Corporation would still create a new site for them at Cameron Toll ^88^.

The example of St. Leonard’s demonstrates the extreme attachment that plotholders form with their land and the anxieties surrounding site closures. Not knowing what would happen from year to year for an extended period led to great uncertainty and stress, yet when the plotholders were given the option of moving to a new, permanent site they instead risked staying together as a community on the site that they loved. The plotholders were also apparently well-educated and well-connected; they knew who to write to and who to ask for help. Their letters and associated letters between the University and the Corporation are incredibly well represented in the *Papers of Victor Webb* and the Edinburgh City Archives. It is likely that other sites in Edinburgh were faced with a similar situation but lacked the skills to fight it. Their thoughts would have gone unrecorded and unrepresented in the archives but that does not mean that they did not experience the same fears and upsets as the St. Leonard’s allotment association. This case study demonstrates that for gardening to truly benefit health and well-being, permanence is needed.

## Discussion

In 2019 community gardens and allotments form the core of Scotland’s urban gardening communities. Charities such as South Seeds and Urban Roots in Glasgow work with volunteers to turn derelict sites into shared gardens. Residents are able to form new relationships with neighbours they might not already know and then work together to improve living conditions in their tenement buildings ^89^.

Most studies on community gardens have been carried out in North America and Australia. The gardens in these places also empower people to overcome local problems, to become places for cultural expression and to learn from each other. They have been shown to improve mental and physical health and have even been praised for having a significantly positive impact on local property prices ^90^. Taking part in gardening also encourages people to become ‘activists’, engaging in local politics and fighting to improve living conditions ^91^. Particularly, community gardens are seen as an answer in times of crisis with attention drawn not just to food production, but perhaps more to the creation of support networks and a sense of purpose and belonging ^92^. There have been two recent studies of Scottish community gardens which focus on the same benefits as North American and Australian researchers; McVey, Nash and Stansbie’s in Edinburgh and Cumbers, Shaw, Crossan and McMasters’ in Glasgow. In 2018 there were 44 community gardens in and around Edinburgh and in 2014 there were 46 community gardens in Glasgow. Both cities are considered to be leading in the number of the community gardens in the UK, along with Bristol and London ^93^.

However, community gardens are often regarded as temporary, created on sites that have been earmarked for developments that have been delayed by recession, just as wartime allotments were created on development sites delayed by the war ^94^. In 2011, Glasgow City Council began a programme called *Stalled Spaces* which supports community groups in creating projects on derelict sites; very often these projects are ‘growing spaces’ and ‘pop-up gardens’ ^95^. Both McVey *et al*. and Cumbers *et al*. stress that taking over derelict sites empowers communities, placing land under their control. Particularly in areas of deprivation, vacant sites can seem hostile or threatening, and creating community gardens positively transforms such spaces ^96^. However, the gardens are reliant on the perpetual delay of developments and funding, which can become a burden. Funding is short-term, not always guaranteed to continue and challenging for applicants who do not have specialist knowledge, therefore putting gardens into ‘a precarious, often uncertain, position’ ^97^. Ironically, gardens can even regenerate derelict sites to such an extent that they drive further development ^98^. Although both McVey *et al*. and Cumbers *et al*. acknowledge the practical difficulties of this, they do not comment on the emotional impact on gardeners when their sites are reclaimed. Community gardens are praised as places to relieve stress and anxiety but the stories of allotment closures in the post-war period, such as West Mains and St. Leonard’s, show that insecurity of tenure causes ** stress and anxiety. Both allotments and community gardens require time to bring land to a good state of cultivation, time within which friendships are formed and communities are built. The St. Leonard’s plotholders had a strong attachment to their site and were unwilling to leave both the land and each other; it is highly likely that today’s community gardeners also have such strong attachments to their sites and to close the gardens would be devastating, yet we persist in creating gardens on land that is only temporarily available.

Community gardens show that there is still a demand for recreational gardening in high-density areas of housing in Scottish cities, but the gardens—similarly to allotments—retain a focus on food production. Much of the literature on community gardening is focused on benefits such as cultural expression and mental health, but it always remains clear that the focus of the gardens is on food. McVey *et al*. state that for most of the people they interviewed, food production was the main motivation, even when it was being used as a tool to address wider social issues ^99^. McVey *et al*. argue that community gardens are an effective way to encourage people to take part in exercise and eat more healthily. The British Medical Association Scotland reports that by 2030, almost 40% of the population of Scotland will be obese. Community gardening can undoubtedly help to avoid this as those involved in the gardens eat 1.4 times more fruit and vegetables than non-gardeners and are 3.5 times more likely to eat the National Health Service’s recommended portion of ‘five-a-day’ ^100^. In a country where only 19% of the population eat this recommended amount, this difference between gardeners and non-gardeners is significant ^101^. Even when SAGS changed their argument to promote allotments for recreation, they never quite lost their insistence that allotments were for food production. There is no reason why recreation and food production need to be separate and, indeed, the literature on community gardens show the two to be closely intertwined in urban gardening. Both allotments and community gardens have a role in healthy eating and it is not just the gardeners who benefit but also the friends and family they share their crops with. However, to be effective, gardens need to be long-term; crops need to be planned and this can only happen in the safe knowledge that your garden is yours for as long as you need it.

## Conclusion

Within Scottish cities, allotments and community gardens can fulfil the post-war arguments put forward by the allotment movement and social researchers such as Thorpe and Jephcott. Urban gardens can prevent loneliness, improve physical and mental health and encourage gardeners to eat more healthily. For gardens to be truly effective places to grow both communities and food we need to learn from the challenges the allotment movement faced in the post-war period and enable them to flourish in permanent, not temporary, spaces.

## Data availability

The collection used for this paper, *The Papers of Victor Webb,* is held by University of Glasgow Archives and Special Collections. The catalogue is available online: https://archiveshub.jisc.ac.uk/search/archives/e81a4437-f0b2-33ad-8229-ef4fc8a0cfdf.

You can access the papers by making an appointment in the Searchroom here: https://www.gla.ac.uk/myglasgow/archives/contact/searchroombookingform/.

A small number of records in this collection are subject to Data Protection legislation as they contain sensitive information; however, access may be given to bona fide researchers and academics. Please contact the Duty Archivist for advice on how to apply for access to these files ( enquiries@archives.gla.ac.uk).

## Footnotes

1 Footnotes beginning UGC are sources from *The Papers of Victor Webb*; footnotes beginning with CA and 3/8 DRT are sources from Edinburgh City Archives; and, footnotes beginning C1 are minutes from Glasgow Corporation meetings held in Glasgow City Archives.

2 Ursula Buchan, *A Green and Pleasant Land: How England’s Gardeners Fought the Second World War* (Northampton: Windmill Books, 2014); Daniel Smith, *The Spade as Mighty as the Sword: The Story of the Dig for Victory Campaign* (London: Aurum Press, 2011).

3 The term community garden is used in a different way in the UK than in the US, where community gardens can be very similar to British allotments. In the UK, ‘community garden’ is used to describe a smaller garden, usually made up of raised beds and communally worked (although sometimes individual raised beds are tended by a single person or one family).

4 Linsay Gray, *Comparisons of Health-Related Behaviours and Health Measures between Glasgow and the Rest of Scotland*, Briefing Paper 7 (Glasgow Centre for Population Health, July 2007); David Walsh, *The ‘Glasgow Effect’ and the ‘Scottish Effect’: unhelpful terms which have now lost their meaning*, < http://www.gcph.co.uk/latest/blogs/641_the_glasgow_effect_and_the_scottish_effect_unhelpful_terms_which_have_now_lost_their_meaning> , 2016; David Walsh, Neil Bendel, Richard Jones and Phil Hanlon, *Investigating a ‘Glasgow Effect’: Why do equally deprived UK cities experience different health outcomes?* (Glasgow Centre for Population Health, April 2010).

5 UGC 222/2/2/5, SAGS Annual Report 1946; UGC 222/2/5/1, Letter McWilliam to SAGS members, 25/02/47.

6 UGC 222/2/5/1, Letter McWilliam to SAGS members, 25/02/47.

7 Andrew Gibb, ‘Policy and politics in Scottish housing since 1945’, in *Scottish housing in the twentieth century*, ed. by Richard Rodger (Leicester: Leicester University Press, 1989), pp. 155–83 (p. 157); Richard Rodger and Hunain Al-Qaddo, ‘The Scottish Special Housing Association and the Implementation of housing policy, 1937–87’, in *Scottish Housing in the Twentieth Century*, ed. by Richard Rodger (Leicester: Leicester University Press, 1989), pp. 184–213 (pp. 188–90).

8 UGC 222/2/3/1, SAGS Conference Report 1947.

9 UGC 222/2/3/1, SAGS Conference Report 1947; UGC 222/2/2/6, SAGS Annual Report 1947.

10 C1/3/117, Sub-Committee on Allotments, 06/01/48; UGC 222/2/5/1, Meeting report 12/01/48.

11 UGC 222/2/5/1, Meeting report 12/01/48.

12 UGC 222/2/5/1/3, Meeting with Woodburn, January 1948.

13 UGC 222/2/2/7, SAGS Annual Report 1948.

14 UGC 222/2/2/9, SAGS Annual Report 1950.

15 UGC 222/2/3/5, SAGS Conference Report 1951.

16 UGC 222/3/5/4/2, Letter Depute Town Clerk to Judge, 28/03/51; UGC 222/3/5/5/12, Letter McWilliam and Judge to McNeil 09/04/51.

17 UGC 222/3/5/5/12, Letter McWilliam to *The Scotsman*, 02/04/51.

18 UGC 222/3/5/5/12, Letter McWilliam to *The Scotsman*, 02/04/51.

19 UGC 222/3/5/4, ‘Allotment Holders’ Protest’, *The Scotsman*, 06/04/51.

20 UGC 222/3/5/5/12, Letter McWilliam and Judge to McNeil, 09/04/51.

21 UGC 222/3/5/5/7, Letter Darling to McNeil, 09/04/51.

22 UGC 222/3/5/4, Letter Depute Town Clerk to Judge, 02/04/51.

23 UGC 222/2/3/5, Webb’s address to SAGS Annual Conference, 21/06/52.

24 Scottish housing is much more closely aligned to the rest of northern Europe than England and Wales, favouring flats over houses. ‘Tenement’ refers to flats mostly built in the late nineteenth to early twentieth century, usually three to four storeys, and including both working-class buildings containing up to twelve flats and upper-class buildings with more spacious flats. In Scotland the word tenement has none of the negative connotations which it does in the US and even the rest of the UK; particularly in larger Scottish cities tenements are a ubiquitous form of housing.

25 UGC 222/2/2/11, SAGS Annual Report 1952.

26 UGC 222/2/3/8, SAGS Conference Report 1954.

27 David Daiches, *Edinburgh* (London: Hamish Hamilton, 1978), p. 227; Michael Fry, *Edinburgh: A History of the City* (London: Macmillan, 2009), p. 355.

28 UGC 222/3/5/2, Edinburgh Corporation Committee Minutes, 15/03/55.

29 UGC 222/3/5/2, Edinburgh Corporation Committee Minutes, 18/10/55.

30 UGC 222/3/5/2, Edinburgh Corporation Committee Minutes, 14/10/52.

31 UGC 222/3/3/3, Judge to EAHA members, 22/08/55.

32 UGC 222/3/5/2, Edinburgh Corporation Committee Minutes, 10/01/56; UGC 222/3/5/2, Edinburgh Corporation Committee Minutes, 03/07/56.

33 UGC 222/3/5/2, Edinburgh Corporation Committee Minutes, 03/07/56; UGC 222/3/5/2, Edinburgh Corporation Committee Minutes, 02/07/57.

34 UGC 222/3/5/2, Edinburgh Corporation Committee Minutes, 18/01/56; UGC 222/3/5/2, Edinburgh Corporation Committee Minutes, 19/02/57.

35 UGC 222/3/5/2, Edinburgh Corporation Committee Minutes, 15/03/55; UGC 222/3/5/2, Edinburgh Corporation Committee Minutes, 13/09/55.

36 UGC 222/2/2/15, SAGS annual report 1956.

37 UGC 222/2/2/15, SAGS Annual Report 1956.

38 UGC 222/2/2/17, SAGS Annual Report 1959.

39 UGC 222/2/2/18, SAGS Annual Report 1960.

40 UGC 222/3/5/4/3, Letter Depute Town Clerk to Secretary of Scottish Development Department, 27/04/64.

41 CA/30/1 DRT 14, Letter Superintendent of Parks to Town Clerk, 29/12/64.

42 CA/30/1 DRT 14, Review of garden allotments.

43 CA/30/1 DRT 14, Letter Superintendent of Parks to the Town Clerk, 29/12/64.

44 UGC 222/2/2/21, SAGS Annual Report 1963.

45 UGC 222/2/2/20, SAGS Annual Report 1962.

46 UGC 222/2/3/16, SAGS Conference Report 1964.

47 UGC 222/2/3/17, SAGS Conference Report 1965.

48 UGC 222/2/2/23, SAGS Annual Report 1965.

49 UGC 222/2/2/23, SAGS Annual Report 1965.

50 UGC 222/2/2/25, SAGS Annual Report 1967.

51 UGC 222/3/5/5/9, Letter Depute Town Clerk to Webb, 16/03/67; CA/30/1 DRT 14, Letter Shade to Town Clerk, 06/05/68.

52 CA/30/1 DRT 14, Letter Shade to Town Clerk, 06/05/68.

53 CA/30/1 DRT 14, Letter Shade to Town Clerk, 06/05/68; CA/30/1 DRT 14, Letter Depute City Chamberlain to Town Clerk, 03/06/68; CA/30/1 DRT 14, Letter Shade to the Town Clerk, 22/06/68; CA/30/1 DRT 14, Letter Director of Parks and Recreation to the Town Clerk, 01/07/68; UGC 222/2/2/26, SAGS Annual Report 1968.

54 CA/30/1 DRT 14, Letter Depute City Chamberlain to Town Clerk, 03/06/68.

55 UCG 222/3/7/3, Harry Thorpe’s Departmental Committee of Inquiry into Allotments Report, October 1969, p. xvi; Lesley Acton, *Growing Space: A History of the Allotment Movement* (Nottingham: Five Leaves, 2015), pp. 145–46.

56 Thorpe, p. 260.

57 Thorpe, pp. 268–77; Acton, p.146.

58 Acton, p. 147; UGC 222/2/2/27, SAGS Annual Report 1969.

59 Thorpe, p. 261.

60 Pearl Jephcott, *Homes in High Flats: Some of the human problems involved in multi-storey housing* (Edinburgh: Oliver and Boyd, 1971), pp. 126–30.

61 Jephcott, p. 111.

62 Pat Rogan ‘Re-Housing the Capital: The crusade against Edinburgh’s slums’ ed. by Miles Glendinning *Re-Building Scotland: The postwar vision 1945–1975’*, (East Linton: Tuckwell Press, 1997), pp. 66–75 (p. 73).

63 3/8 DRT 14, Letter Smith to Depute Town Clerk, 06/08/49.

64 3/8 DRT 14, Letter Smith to Depute Town Clerk, 06/08/49.

65 3/8 DRT 14, Letter Superintendent of Parks to Depute Town Clerk, 11/08/49.

66 3/8 DRT 14, Letter University of Edinburgh solicitors to Town Clerk, 14/10/49.

67 3/8 DRT 14, Letter Town Clerk to University of Edinburgh solicitors, 18/10/49; 3/8 DRT 14, Letter Depute Town Clerk to City Chamberlain, City Architect, Superintendent of Parks, 21/10/49.

68 3/8 DRT 14, Letter Depute Town Clerk to Councillor Waterston, Councillor Earsman, W. Smith, 30/11/49; 3/8 DRT 14 Letter Depute Town Clerk to John Cook, solicitor, 15/12/49; 3/8 DRT 14, Letter Depute Town Clerk to allotment tenants, 29/12/49; 3/8 DRT 14, Letter Depute Town Clerk to Smith, 29/12/49.

69 3/8 DRT 14, Letter Smith to Depute Town Clerk, 29/07/50.

70 3/8 DRT 14, Letter Smith to Depute Town Clerk, 29/07/50.

71 3/8 DRT 14, Letter Smith to Depute Town Clerk, 09/12/50.

72 3/8 DRT 14, Letter Smith to Depute Town Clerk, 18/05/51; 3/8 DRT 14, Letter Smith to Depute Town Clerk, 03/05/52; 3/8 DRT 14, Letter Smith to Depute Town Clerk, 15/04/53.

73 UGC 222/3/2/3, Letter from committee to Secretary of State, 29/07/53.

74 UGC 222/3/5/2, Edinburgh Corporation Committee Minutes, 09/03/54.

75 UGC 222/3/2/3, Letter Appleton to Smith, 05/02/54.

76 3/8 DRT 14, Letter Town Planning Officer to Town Clerk, 08/03/54.

77 3/8 DRT 14, Letter Superintendent of Parks to Town Clerk, 04/02/54; 3/8 DRT 14, Letter Town Clerk to Smith, 26/03/54.

78 3/8 DRT 14, Letter Smith to Depute Town Clerk, 25/05/54.

79 3/8 DRT 14, Letter Smith to plotholders, 19/10/54.

80 3/8 DRT 14, Letter Smith to Depute Town Clerk, 01/10/55.

81 3/8 DRT 14, Letter Smith to Depute Town Clerk, 01/10/55.

82 UGC 222/3/5/2, Edinburgh Corporation Committee Minutes, 18/10/55.

83 UGC 222/3/5/2, Edinburgh Corporation Committee Minutes, 18/10/55.

84 3/8 DRT 14, Letter Town Planning Officer to Depute Town Clerk, 21/10/55.

85 UGC 222/3/2/3, Letter from University of Edinburgh Administrative Assistant to Webb, 25/11/55.

86 3/8 DRT 14, Minute of meeting at Edinburgh University, 30/11/55.

87 UGC 222/3/2/3, Letter from Smith to Webb, 03/11/56.

88 UGC 222/3/4, Letter Smith to Webb, 28/02/64.

89 Owen Duffy, ‘Tackling Glasgow's substandard homes with peas, beetroot and thermal imaging’, *The Guardian*, 12 ^th^ September 2012, < http://www.theguardian.com> [Accessed 15/11/2013]; Urban Roots, < http://www.urbanroots.org.uk> [Accessed 6th January 2014].

90 Donna Armstrong, ‘A survey of community gardens in upstate New York: Implications for health promotion and community development’, *Health and Place* 6 (2000), pp. 319–27 (pp. 319–327); Lauren E Baker, ‘Tending cultural landscapes and food citizenship in Toronto’s community gardens’, *Geographical Review* 94:3 (2004), pp. 305–25 (pp. 319–22); Walter W Furness and Courtney M Gallaher, ‘Food access, food security and community gardens in Rockford, IL’, *Local Environment* 23:4 (2018), pp. 414–430 (p. 414); Leigh Holland, ‘Diversity connections in community gardens: a contribution to local sustainability’, *Local Environment: The International Journal of Justice and Sustainability* 9:3 (2004), pp. 285–305 (p. 287); Ioan Voicu and Vicki Been, ‘The Effect of Community Gardens on Neighbouring Property Values’, *Real Estate Economics* 36:2 (2008), pp. 241–83 (p. 243); Pierre Walter, ‘Theorising community gardens as pedagogical sites in the food movement’, *Environment Education Research* 19:4 (2012), pp. 521–39 (p. 524).

91 Baker, p. 305; Efrat Eizenburg, ‘Actually Existing Commons: Three Moments of Space of Community Gardens in New York City’, *Antipode* 44:3 (2012), pp. 764–82 (pp. 777–78).

92 Bethaney Turner, Joanna Henryks and David Pearson, ‘Community gardens: sustainability, health and inclusion in the city’, *Local Environment: The International Journal of Justice and Sustainability*, 16:6 (2011), pp. 489–92 (p. 490).

93 David McVey, Robert Nash and Paul Stansbie, ‘The motivations and experiences of community garden participants in Edinburgh, Scotland’ in *Regional Studies, Regional Science* 15:1 (2018), pp. 40–56 (p. 41); Andrew Cumbers, Deirdre Shaw, John Crossan, Robert McMaster ‘The Work of Community Gardens: Reclaiming Place for Community in the City’, *Work, Employment and Society* 32:1 (2018), pp. 133–149 (p. 134, p. 136).

94 < www.centralscotlandgreennetwork.org> [Accessed 26/02/2019].

95 < www.glasgow.gov.uk> [Accessed 26/02/2019].

96 McVey *et al.*, p. 52–53; Cumbers *et al.*, p. 139–40.

97 McVey *et al*., p. 54; Cumbers *et al.*, p. 146.

98 Cumbers *et al*., p. 135.

99 McVey *et al*., p. 50.

100 McVey *et al*., p. 42; < https://www.nhs.uk/live-well/eat-well/why-5-a-day/> [Accessed 13/03/2019].

101 Jenny Mollison, Judy Wilkinson and Rona Wilkinson, *Raising Spirits: Allotments, well-being and community* (Edinburgh: Argyll Publishing, 2015), p. 18.
