# Supplementary material for: A place to grow: Well-being and activism on Edinburgh’s post-war allotments and how this can inform urban gardening in Scotland today
Source: Wellcome Open Res. 2019 Apr 17;4:72. [Version 1] doi: 10.12688/wellcomeopenres.15216.1 (PMC6544128; doi:10.12688/wellcomeopenres.15216.1)
Supplement: Peer review history

# Peer review: Reviewer response for version 1

**DOI:** 10.21956/wellcomeopenres.16605.r35348

**Published:** 2019-05-29

**Authors:** Lesley Acton

**Affiliations:** 1 Independent Researcher, Manchester, NH, USA

Hannah Connelly has based her research on papers formally held by the Scottish Allotments and Gardens Society, which now form part of the Special Collections and Archive of the University of Glasgow. 

 This paper juxtaposes the loss of allotments sites today with that in the post WWII period in order to answer the following questions:

- How did SAGS campaign for the survival of allotments in the post-war period and how did this shape the development of the society?
- What was the impact of allotment closures on plotholders in the post-war period and how can this impact inform the use of community gardens today?
- Is there a need for permanence on allotments and community gardens to truly benefit health and well-being?

By and large, these questions have been answered, although some further research would give greater depth to the paper. 

 There is a nice snapshot on page 3 of the housing situation in Scotland at the end of WWII. The dire need for housing eclipsed the realisation by more forward thinking urban planners that green spaces, such as allotments and community gardens, were, and indeed are, important in creating and maintaining a community. 

 The author makes the point that security of tenure was, and still is, vital in making community gardens successful. I think this paper would benefit from a fuller explanation as to why security of tenure and tidiness, which is mentioned several times throughout the paper, are so important. There are many other factors that contribute to the success of a community garden that are touched on (fencing/vandalism, distance to home, horticultural potential of soil, health benefits, etc.,).  These should be more fully explained, as should the tension between Scottish government and SAGS (page 3, column 1), because all these same factors contribute to the success, or otherwise, of community gardens today. Such a comparison would add weight to the discussion and conclusion. 

 The explanation about the difference between tenements in Scotland with those in England and Wales should not be relegated to a footnote. That Scottish tenements covered a broad range of housing types is important in understanding how tenement dwellers comprised a wide spectrum of socio-economic groups. Accordingly, some may have gardened for economic reasons, while others gardened for recreation with ‘food benefits.’  Knowing what motivates gardeners allows a community garden provider to better manage stakeholder expectations. 

 Top of page 5, column 1, ‘local authorities continued to close sites for housing development.’ Are there more data to contextualise that assertion? 

 Page 5, column 2, discusses that sites continued to close throughout the 1960s. What other factors were at play here other than just sites being built over? 

 Page 6, column 1, ‘tidiness on plots to demonstrate that they were fully cultivated and actively in use, but this would have restricted differences in gardening methods’. How so? 

 ‘Tidy sites were more likely to be saved by the Edinburgh Corporation, which meant sites in wealthier areas might have been more likely to be saved where this risk could be taken without fear of financial loss.’ This statement appears to assume that wealthier plotholders did not have the same emotional attachment to their sites as those from lower economic groups. Is this the case? 

 Page 7, column 1, ‘Leisure Garden’. A discussion of the Thorpe Report’s Leisure Garden concept ^1^ would add to the context here. 

 Page 8, column 2, ‘One of the difficulties in allotment history is that it is not always possible to see what plotholders themselves thought or felt about site closures.’ Also, page 10, column 2, ‘the extreme attachment that plotholders form with their land’. Local newspapers and minutes of council meetings might provide some evidence of plotholder views and feelings. Same paragraph, ‘well-educated and well-connected’, what is the supporting data? 

 Page 11, column 2, ‘it is highly likely’. Supporting primary sources are needed. Interviewing plotholders whose land is under threat would give this paper greater significance. 

 I enjoyed reading this paper, and I am delighted that Wellcome has chosen to fund this project and add to the growing body of evidence of the many benefits provided by community gardens.

I have read this submission. I believe that I have an appropriate level of expertise to confirm that it is of an acceptable scientific standard.

## References

[1] ThorpeH: Departmental Committee of Inquiry into Allotments Cmnd.4166.1969; Reference source

# Peer review: Reviewer response for version 1

**DOI:** 10.21956/wellcomeopenres.16605.r35347

**Published:** 2019-05-13

**Authors:** Nina J. Morris

**Affiliations:** 1 University of Edinburgh, Edinburgh, UK

Drawing principally on the collected papers of Victor Webb, an Edinburgh allotment-keeper heavily involved in the Scottish Allotments and Gardens Society and the Scottish Allotments Scheme for the Unemployed, this paper juxtaposes the post-war closure of allotments in Edinburgh (and beyond) with the creation of contemporary community gardens on land earmarked for future development. In particular, the paper focuses on the impact (and the threat of) such reclamation had/might have on plotholders’ mental health and well-being. As such, I believe the paper addresses a demonstrable gap in, and makes a valuable contribution to, current debates surrounding urban greenspace, cultivation, and recreation. The paper is scientifically sound, however, responses to the following comments/questions would provide useful clarification.

 I think it would be useful to briefly explain the nature ‘archive-driven’ (p.2, column 1) research for those who might not be familiar with this type of approach.

 In the Introduction (p.2, column 1) you state that ‘when the war ended this land was reclaimed’. Were the plotholders advised of the short-term nature of the let when they took on the allotments? Are there allotments remaining today which were not subject to reclamation and why might this have been the case?

 Have local authorities and charities only recently started to encourage residents to create community gardens (p.2, column 1) or does this date back some years? Some sense of a timeframe would be useful.

 It is not completely clear from the ends of paragraphs 1 and 2 (p.2, columns 1 and 2) what the main focus of the paper will be. Some refinement of the paper’s purpose would be useful.

 You state that the paper will consider ‘what was the impact of allotment closures on plotholders in the post-war period and how this can impact inform (sic) the use of community gardens today’ (p.2, column 2). The sentence construction needs attention here but also, is it the ‘use’ that you are interested in informing or the statutory rights of the plotholders and/or tenancy agreements/landownership?

 Did insecurity of tenure, etc. totally ‘negate’ (p.2, column 2) the positive experiences plotholders gained?

 Should it be ‘the new society began its campaigns’ or ‘campaign’ (p.3, column 1)?

 Towards the end of paragraph 1 (p.3, column 1) it would be useful to know how many plots were free/had been abandoned in light of Robert Fraser's claims. Was he basing his view on statistical or anecdotal evidence?

 The sentence beginning ‘Although rationing’ (p.4, column 2) seems a little repetitive.

 It is not clear to me what ‘would have restricted differences in gardening methods’ means (p.6, column 1). Could you explain?

 Presumably the extensive waiting lists for allotments in Edinburgh today is also proof of demand (p.8, column 1)?

 Are all community gardens in a precarious position (p.8, column 2)? If so, are they precarious for the same reasons?

 Given the focus of the paper is on Edinburgh’s post-war allotments, are there any charities/groups working with Edinburgh-based communities to renovate derelict sites (p.10, column 2)?

 It would be useful to know how many of the 44 community gardens in Edinburgh (and allotments) are currently on ‘temporary’ or relatively short-lease sites (p.11, column 1).

 Some community gardens now have short-term lease built into their design (i.e. they are designed to be moved when the land is required by the developer) but it is not clear if this has been taken into account in your discussion.

 On the whole, I found this an interesting and thought-provoking paper on a highly relevant topic which will be of interest to not just to academics working within the disciplines of geography (my own discipline) and history but also in the related fields of health and well-being, landscape architecture, and planning.

I have read this submission. I believe that I have an appropriate level of expertise to confirm that it is of an acceptable scientific standard.
